# Greater Physiological and Behavioral Effects of Interrupted Stress Pattern Compared to Daily Restraint Stress in Rats

**DOI:** 10.1371/journal.pone.0102247

**Published:** 2014-07-11

**Authors:** Wei Zhang, Andrea Hetzel, Bijal Shah, Derek Atchley, Shannon R. Blume, Mallika A. Padival, J. Amiel Rosenkranz

**Affiliations:** The Chicago Medical School, Rosalind Franklin University of Medicine and Science, North Chicago, Illinois, United States of America; University of São Paulo, Brazil

## Abstract

Repeated stress can trigger a range of psychiatric disorders, including anxiety. The propensity to develop abnormal behaviors after repeated stress is related to the severity, frequency and number of stressors. However, the pattern of stress exposure may contribute to the impact of stress. In addition, the anxiogenic nature of repeated stress exposure can be moderated by the degree of coping that occurs, and can be reflected in homotypic habituation to the repeated stress. However, expectations are not clear when a pattern of stress presentation is utilized that diminishes habituation. The purpose of these experiments is to test whether interrupted stress exposure decreases homotypic habituation and leads to greater effects on anxiety-like behavior in adult male rats. We found that repeated interrupted restraint stress resulted in less overall homotypic habituation compared to repeated daily restraint stress. This was demonstrated by greater production of fecal boli and greater corticosterone response to restraint. Furthermore, interrupted restraint stress resulted in a lower body weight and greater adrenal gland weight than daily restraint stress, and greater anxiety-like behavior in the elevated plus maze. Control experiments demonstrated that these effects of the interrupted pattern could not be explained by differences in the total number of stress exposures, differences in the total number of days that the stress periods encompased, nor could it be explained as a result of only the stress exposures after an interruption from stress. These experiments demonstrate that the pattern of stress exposure is a significant determinant of the effects of repeated stress, and that interrupted stress exposure that decreases habituation can have larger effects than a greater number of daily stress exposures. Differences in the pattern of stress exposure are therefore an important factor to consider when predicting the severity of the effects of repeated stress on psychiatric disorders.

## Introduction

Repeated stress exacerbates depression and anxiety, and can trigger post-traumatic stress disorder. The effects of stress depend on a range of factors, and are believed to be proportional to the severity and frequency of the stress exposure [1]–[12]. However, the pattern of stress exposure may also play a role in its effects [13], [14]. Common patterns of stress exposure include 1) continuous over an extended period of time (chronic), 2) daily acute episodes over an extended period (daily), or 3) sporadic acute episodes over an extended period. Two types of sporadic exposures include intermitent, when the stressor is not a frequent occurrence over a given period of time, or interrupted, when the stressor occurs frequently over a given period of time with occassional interuptions. Stress exposure in humans is often in an interrupted pattern. A classic example is 5 day work-related stress interspersed with 2 day weekends. There have been few studies to test whether interrupted stressors exert the same effects on physical and behavioral manifestations of repeated stress.

Repeated exposure to the same stressor leads to gradual reduction of the stress response to that stressor (homotypic habituation; [10], [15]–[19]). Behavioral and endocrine habituation to a stressor is a key component of adaptation, and contributes to coping strategies, which in turn are associated with vulnerability to the effects of stress [20], [21]. The degree of habituation depends on the severity of the stressor [10]–[12], [22]–[24], with much less habituation to severe stressors [11], [25]–[29]. This reduced habituation parallels greater adverse effects of severe stress in rodent models of anxiety and depressive behavior. Furthermore, exposure to variable distinct stressors, which precludes homotypic habituation, leads to greater impact on several behavioral and physiological measures than repeated exposure to the same stressor [30].

Previous studies demonstrate that the frequency of stress exposure is a critical element in habituation [31]–[33]. The regularity of the pattern of stress exposure influences the rate of habituation and effects on hypothalamic-pituitary-adrenal (HPA) indices [13], [14]. An understanding of the relationship between stress exposure and behavioral/physiological outcomes can provide insight into the relative impact of different stress exposure patterns, and the functional role of habituation in the behavioral outcome. However, few studies have compared habituation to distinct patterns of exposure, such as daily and interrupted stressors, and their relation to behavior. For instance, one might expect that daily exposure to stressors would lead to greater effects on anxiety behavior than interrupted exposure. However, if daily exposure allows more rapid habituation than interrupted exposure, the opposite might be predicted.

This study will hold stressor type, duration and intensity constant, and test whether interrupted stress exposure (and associated differences in habituation) leads to greater effects on anxiety-like behavior. Body weight, adrenal gland weight, plasma corticosterone, and anxiety-like behavior in the elevated plus maze were measured to assess the acute and cumulative effects of repeated restraint in a daily exposure or an interrupted exposure pattern. These studies have signficance for the relationship between stress exposure and adverse effects in psychiatric disorders. However, the difference between daily and interrupted stressors, and the role of habituation in mediating these actions are not known. Further insight into the role for habituation in the responses to stress can lead to novel, clinically useful predictive ability for the effects of repeated stress, as well as potential therapeutic approaches to target habituation processes.

## Materials and Methods

All experiments were approved by the Institutional Animal Care and Use Committee of Rosalind Franklin University (protocol #11-47 and 13-10), and followed the Guide for the Care and Use of Laboratory Animals (National Research Council, 2011). Efforts were made to minimize animal suffering and to reduce the number of animals used. At the end of the study, rats were euthanized by overdose of ketamine/xylazine followed by decapitation.

Male Sprague-Dawley rats (Harlan; age 8–9 weeks at arrival, total n = 536 rats) were group housed (2–3/cage) in a controlled climate animal facility. The housing room had a 12∶12 light∶dark schedule and food and water were available ad libitum. Restraint stress was performed by placement of a rat in a restraint hemi-cylinder for 20 minutes per session. The restraint device was a transparent acrylic cylinder with flattened bottom, with holes for ventilation (dimensions 8″×3.25″). A control group was handled in the same manner as the restraint group, except that they remained in a transparent transport cage with bedding, instead of placement in a restraint cylinder.

### Repeated restraint patterns rationale

**Figure 1:**
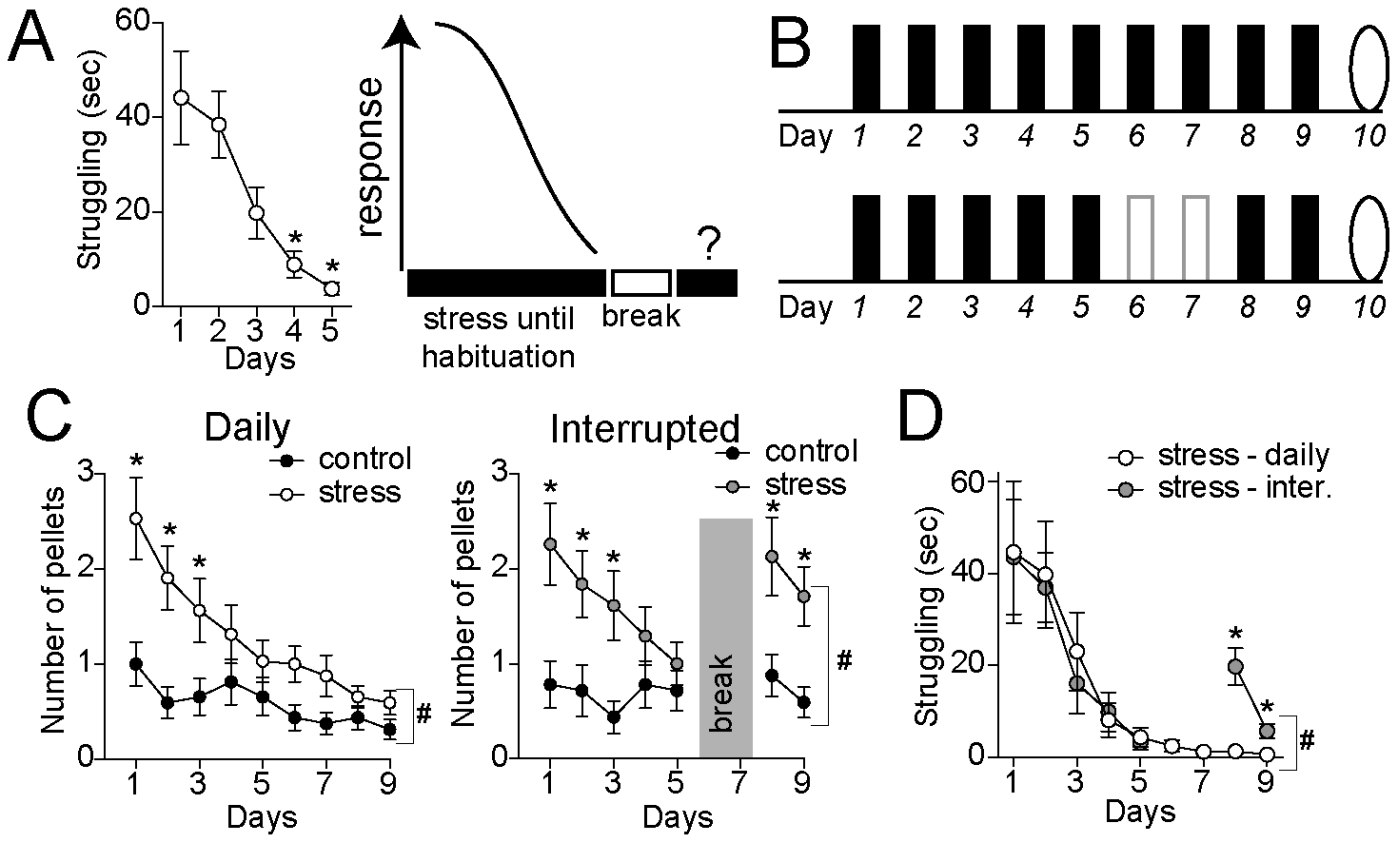
Reduced habituation of fecal pellet production during the interrupted restraint. A) The interrupted restraint protocol was designed to test the effects of an interruption placed shortly after the emergence of habituation to a repeated stressor. Emergence of habituation was initially measured as a significant reduction in struggling behavior during restraint over the course of 5 days. B) In these studies, daily stress was compared to an interrupted stress pattern where a two day interruption was placed after 5 days of daily stress. The solid black bars indicate a stress day, the open bars indicate an interruption day, while the oval indicates the test day. C) Fecal pellet production was counted at the end of each daily stress or control session. Significant habituation of pellet production to control levels was observed by the 5^th^ day of daily restraint stress. This habituation persisted through the remaining stress sessions in the daily restraint group. Significant habituation of pellet production was also observed in the interrupted restraint group by the 5^th^ day, prior to the interruption. After the interruption, pellet production increased, and was significantly different than controls. D) The interrupted stress group displayed significantly greater pellet production after the interruption compared to the daily stress group. * indicates p<0.05 in post hoc tests comparing to the first day of restraint in a one-way ANOVA (A), or comparing control and stress (C) or daily and interrupted stress (D) after significance in a two-way repeated measures ANOVA. Here and in all subsequent figures, inter = interrupted pattern, and daily = daily pattern. # indicates significant Pattern x Day interaction in a two-way ANOVA.

To compare interrupted and daily stress, the most basic protocol was selected; application of a single interruption over the course of daily stress exposure, applied shortly after a significant habituation was observed. In preliminary experiments, consecutive daily exposures to restraint was examined. After 5 consecutive daily exposures to restraint (days 1–5; Figure 1) a reduction of the acute response to restraint was observed, consistent with habituation in other studies (e.g. [34], [35]). Therefore, this was chosen as the time to impose an interruption. The short interruption was used as a tool to stall or reverse habituation and test the difference between the effects of daily exposure and interrupted exposure on habituation. In preliminary experiments, an interruption of 1 day was not enough to recover from habituation (as measured by pellet production and body weight), similar to previous findings [36]. An interruption of two days (days 6–7; Figure 1) was verified to allow regain of body weight to control levels and re-emergence of struggling during restraint. Two additional days of restraint after the interruption were chosen to measure recovery of the response to restraint (day 8), and subsequent habituation after the interruption (day 9). This pattern was compared to 9 consecutive days, 7 consecutive days, 2 consecutive days or single restraint sessions.

### Measures during restraint

#### Measurement of corticosterone

To measure corticosterone (CORT), one of the primary stress hormone in rats, blood samples were collected by tail nick during the 20^th^ minute of restraint or control procedures. This sampling occurred between 9–11 AM, when levels are near their basal diurnal state. Blood (approximately 300 µL) was placed in a tube containing heparin, and immediately placed on ice, then centrifuged at 3200 rpm for 5 min at 4°C. Clear plasma was pipetted out, put into tubes and frozen at −20°C until analysis. Samples were analyzed by enzyme immunoassay for corticosterone (Corticosterone EIA kit, Cayman Chemicals), following the manufacturer's suggested protocol. Absorbance was measured and quantified against known concentrations of corticosterone (405 nm; Novostar multiwell plate reader, BMG LabTechnologies, Ortenberg, Germany). All samples were run in duplicate. Corticosterone concentration in samples were interpolated from an exponential fit to percent bound ÷ maximal bound (%B/Bmax) curve from known concentrations and expressed as pg/mL. For calculation, absorbance measures of blank controls and non-specific binding controls were subtracted out from all values.

#### Measurement of fecal boli

The total number of fecal boli during restraint or control procedures was quantified by counting individual boli in the restraint cylinder after restraint or in the transport cage of control rats.

#### Measurement of struggling

The amount of time spent struggling was quantified. This measure was comprised of time spent chewing on the restraint apparatus, attempts to back out of the apparatus, attempts to turn around in the apparatus, and any other behavior that included gross movement of limbs. These behaviors were scored manually with a digital stopwatch by trained observers during the restraint session. The stopwatch was activated and deactivated with the initiation and cessation, respectively, of struggling behaviors. In initial experiments, scoring was confirmed offline from a recorded video until >85% concordance between observers was obtained.

### Cumulative effects of restraint

#### Body weight

Body weight was measured daily prior to restraint or control procedures.

#### Adrenal gland weight

Adrenal glands were excised after euthanasia. Both adrenal glands were weighed while wet. Adrenal gland weight was normalized to body weight (mg/g of body weight).

### Elevated plus maze

Animals were tested in the elevated plus maze (EPM) one day after the final restraint/control handling session. The EPM (Scientific Design, Pittsburgh, PA) consisted of four arms: two open arms (width×length 4.25″×19.75″) and two closed arms (width×length×wall height 4.25″×19.75″×18″). Each arm was attached to a sturdy leg, elevated 32″ from the ground. Animals were placed at the junction of the four arms, facing the open arm opposite the experimenter. Animal behavior was recorded for 5 min and analyzed by a personal computer (Dell E6500, Dell, Round Rock, TX) running video-tracking software (Any-Maze, Stoelting, Wood Dale, IL). The time spent on open arms was measured and used as an index of anxiety-like behavior. In addition, the total number of arm entries was measured and used as an indicator of locomotor activity.

### Analysis

Raw data was tested for normality of distribution (D'Agostino and Pearson normality test) and homogeneity of variance (Bartlett's test). If data sets were found to display non-normal distributions, non-parametric analysis was used (Kruskal-Wallis with post-hoc Dunn's tests). Data was analyzed across days with two-way repeated measures ANOVAs. Treatment pattern x stress group comparisons were performed with two-way ANOVAs. If significant effects were observed, post-hoc comparisons were performed as appropriate, with Bonferroni corrections for multiple comparisons. Two-tailed unpaired t-tests were used for specific planned comparisons. A p value of less than 0.05 was considered statistically significant. All data is presented as mean ± SEM.

## Results

### Effects of daily and interrupted repeated restraint

In preliminary studies it was observed that rats avoided entry into the restraint cylinder and once in the cylinder displayed struggling behavior for several minutes in apparent attempts to escape the cylinder on the first days of restraint. However, by the 5^th^ session of consecutive daily restraint, rats often went into the restraint cylinder with little direction, did not struggle, and appeared to be in a passive coping state, similar to other studies [34]. When quantified in a subset of rats, there was a significant reduction of struggling across days (Fig. 1A; n = 24; one-way repeated measures ANOVA, p<0.0001, F(4,92) = 17.4; significant differences between Day 1 and Days 4–5, p<0.05 in post-hoc comparisons with Bonferroni corrections). This is consistent with homotypic habituation to a repeated stressor. Production of fecal pellets is an additional quantifiable measure of responsiveness to stressors [37]–[39]. There was a reduction in the number of fecal pellets produced during restraint over consecutive restraint sessions (Fig. 1C; control n = 32, restraint n = 32; two-way repeated measures ANOVA, Stress treatment x Day, significant main effect of Day p<0.0001, F(8,496) = 8.87, significant main effect of stress p = 0.0005, F(1,62) = 13.3; significant difference between control and stress groups at days 1,2,3, p<0.05 in post-hoc comparisons with Bonferroni corrections).

**Figure 2:**
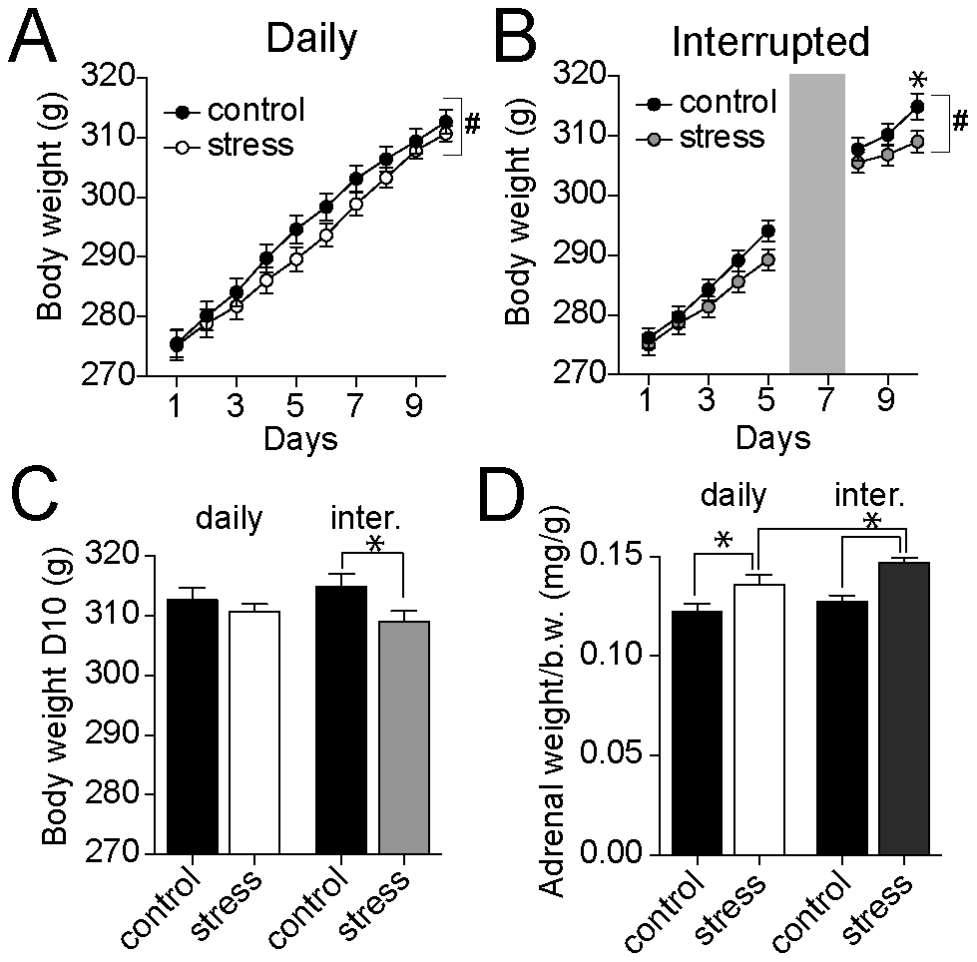
Interrupted stress has greater impact on body weight and adrenal gland weight. A) Weight gain over time in the daily stress group compared to the daily control group was measured. There was a significant Day x treatment pattern (2-way repeated measures ANOVA). However, body weight on any individual day was not significantly different between groups in post hoc comparisons after correction for multiple comparisons. B) Interrupted stress led to a transient difference in body weight that recovered during the interruption, but reappeared after the resumption of stress. C) When measured one day after the last stress or control session, daily stress did not have a significant effect on body weight, while interrupted stress significantly decreased body weight. D) Adrenal gland weight was increased by daily or by interrupted stress (normalized to body weight displayed here). The effect of interrupted stress on adrenal gland weight was significantly greater than daily stress. * indicates p<0.05 in post hoc tests after significance in a two-way repeated measured ANOVA (B) or two-way ANOVA (C, D). # indicates significant Pattern x Day interaction in a two-way ANOVA.

Previous studies have demonstrated transient weight loss associated with repeated mild stress [8], [40]–[43]. Here too, repeated stress transiently decreased the weight gain curve compared to control rats (Fig. 2A, days 5–7). However, rats regained weight close to control levels over subsequent days of repeated restraint, further indicative of habituation to the stressor (Fig. 2A). This difference in weight gain is transient, but significant (daily control n = 40, daily restraint n = 44; two-way repeated measures ANOVA, Stress treatment x Day, significant main effect of Day p<0.0001, F(9,738) = 881.9; no significant main effect of stress p = 0.32, F(1,82) = 0.99; significant Treatment x Day interaction p = 0.0002, F(9,738) = 3.56).

Discontinuation after several exposures to a moderate stressor can stall or perhaps reverse habituation. However, with a large number of repeated exposures to a moderate stressor, the reversal of habituation can take weeks [44]–[48]. To test whether robustness of the stress response can be restored after habituation to a short course of restraint stress, a break in continuity of the daily restraint sessions was applied (Fig. 1A,B; producing an interrupted pattern). The break in the restraint procedure was applied after day 5 of restraint, a point at which habituation is observable in struggling behavior and the production of fecal pellets (Fig. 1A,C). After imposing a two day break from restraint, rats were noticeably more resistant to enter into the restraint cylinder, and struggling behavior re-emerged during restraint (Fig. 1D; daily restraint n = 13, interrupted restraint n = 11; two-way repeated measures ANOVA, Stress pattern x Day, significant main effect of stress p<0.0001, F(6,132) = 15.5; significant difference between daily and interrupted stress groups at days 8 and 9, p<0.05 in post-hoc comparisons with Bonferroni corrections). The number of fecal pellets during restraint was also significantly increased (Fig. 1C; interrupted control n = 31, interrupted restraint n = 32; daily control n = 32, daily restraint n = 32; two-way repeated measures ANOVA, Stress Treatment x Pattern, significant main effect of stress p<0.0001, F(13,861) = 8.8; significant main effect of pattern p = 0.05, F(1,861) = 3.9; significant stress x pattern interaction p = 0.03, F(13,861) = 1.81; significant difference between daily stress and interrupted stress groups at days 8 and 9, p<0.05 in post-hoc comparisons with Bonferroni corrections).

Furthermore, during the two day interruption restraint-stressed rats regained weight, but upon re-initiation of stress after the 2 day interruption, a difference in weight between the control and stress groups reemerged (Fig. 2B; interruption control n = 78, interruption stress n = 71; two-way repeated measures ANOVA, Stress pattern x Day, significant interaction p<0.0001, F(7,1022) = 8.5; Day 10 body weight p<0.05, post-hoc comparisons with Bonferroni corrections). Body weight was significantly different between control and stress in interrupted stress groups (Fig. 2C; control = 314.8±2.2 g, stress = 309.0±1.8 g) but not in daily stress groups (control = 312.6±2.1 g, stress = 310.6±1.3 g, one-way ANOVA of weight at Day 10, Stress treatments x Treatment pattern, p = 0.041, F(3,211) = 2.80, p>0.05, post-hoc comparisons with Bonferroni corrections). These data indicate that significant habituation occurred over the first 5 days of repeated stress and that the stress response displayed some recovery after a 2 day interruption. Thus, this is a good model to test the outcomes of an interrupted pattern of stress that modulates habituation.

A hallmark outcome of the cumulative effects of stress is an increase in adrenal gland weight and function [25], [49]–[53]. Adrenal gland weight was measured here, and adrenal gland function was tested below. Both repeated stress in a daily pattern and interrupted pattern led to an increase of adrenal gland weight when normalized to body weight (Fig. 2D; daily control n = 19, daily stress n = 22, interrupted control n = 50, interrupted stress n = 57; two-way ANOVA of normalized adrenal weight at Day 10, Stress treatments x Treatment pattern, main effect of stress p<0.0001, F(1,144) = 19.0, main effect of pattern p = 0.03, F(1,144) = 4.8, significant stress x pattern interaction p = 0.04, F(1,144) = 4.2; significant difference between control and stress in both treatment patterns, p<0.05 in post-hoc comparisons with Bonferroni corrections), or when raw values were examined (daily control = 39.4±0.8 g, daily stress = 42.1±1.4 g, interrupted control = 41.3±0.7 g, interrupted stress = 46.9; two-way ANOVA of adrenal weight at Day 10, Stress treatments x Treatment pattern, main effect of stress p<0.0001, F(1,144) = 20.2, main effect of pattern p = 0.0004, F(1,144) = 13.4; significant stress x pattern interaction p = 0.04, F(1,144) = 4.3; significant difference between control and stress in both treatment patterns, p<0.05 in post-hoc comparisons with Bonferroni corrections). However, the effects of interrupted stress were greater than the effects of daily stress (Fig. 2D; adrenal weight significantly greater in interrupted stress group compared to daily stress group, p<0.05 in post-hoc comparisons with Bonferroni corrections).

**Figure 3:**
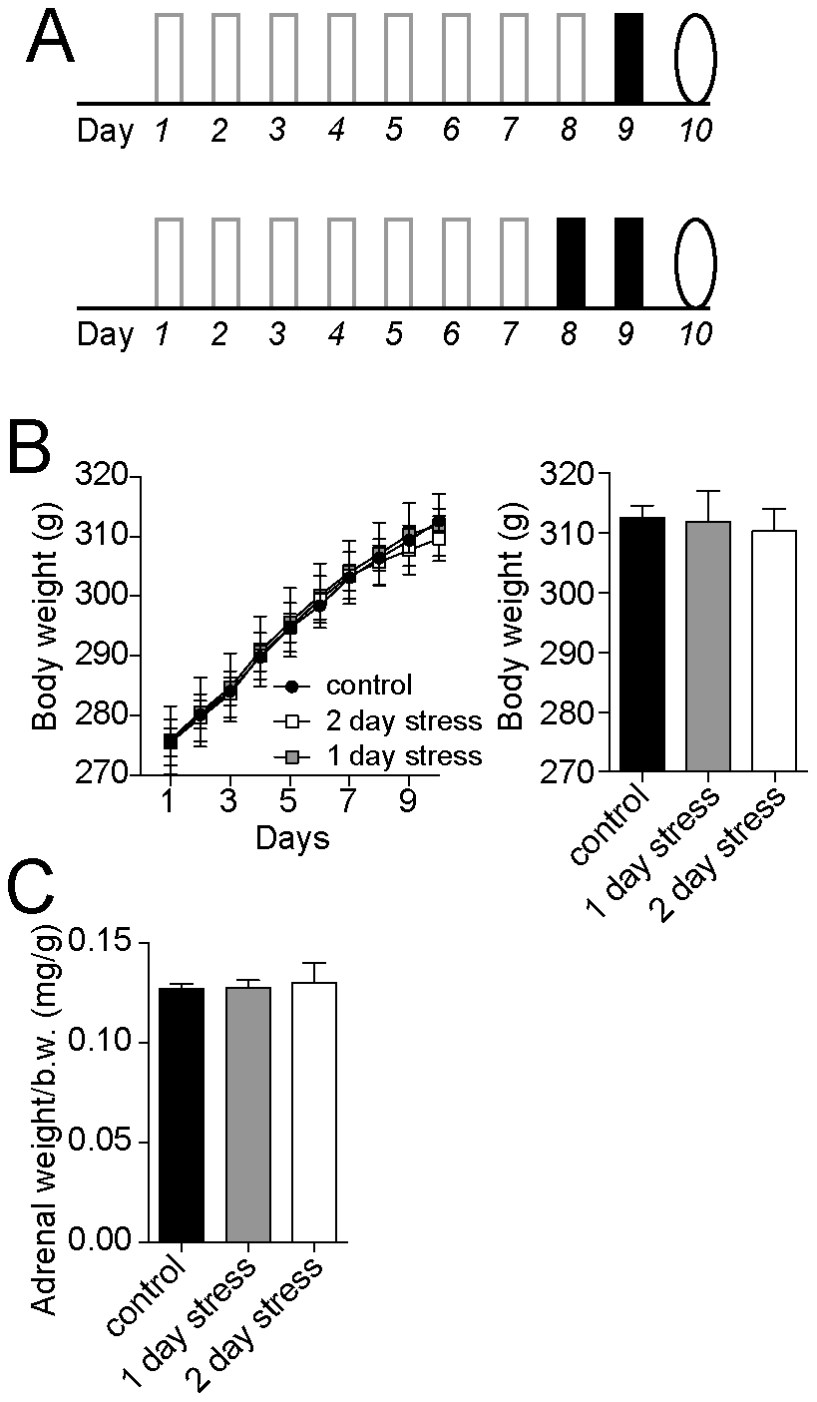
One or two day stress does not have significant effects. A) To control for effects due to a single stress or two days of stress, and not due to the cumulative effects of stress, the effects of one (top) or two days (bottom) of stress was examined. Solid bars indicate the day of stress, open bars indicate days of no stress, oval indicates the test day. B) Neither one nor two days of stress had any significant effects on body weight over the course of the experiment, nor on body weight one day after the final stress, compared to control handling. C) Neither one nor two days of stress had any significant effects on adrenal gland weight.

A single stress can be a potent stimulus. To test whether the effects observed here could be attributed to a lasting effect reflecting only the last restraint episode, the effects of a single restraint were examined (Fig. 3A). A single stress did not cause a significant effect on body weight measured one day after restraint (Fig. 3A,B; control n = 40, 1 day stress n = 30; two-way repeated measures ANOVA, Treatment pattern x Day, no significant main effect of Treatment pattern p = 0.85, F(1,68) = 0.036; no significant difference of body weight on day 10, two-tailed t-test p = 0.50, t = 0.68, df = 49). There was also no significant effect of a single restraint on adrenal gland weight measured one day after a single restraint stress (Fig. 3C; control 39.8±0.8 g, n = 30, stress 40.7±0.9 g, n = 30; two-tailed t-test p = 0.84, t = 0.203, df = 68). Therefore, it is unlikely that the effects observed here could be attributed to the final restraint episode.

### Do differences reflect only the final two days of restraint?

It is possible that the difference in weight and fecal pellets between daily and interrupted stress patterns simply reflects the effects of the final two days of restraint after the interruption, and is not dependent on the interrupted stress history. Several pieces of data argue against this possibility. In the stress pattern applied above, the effects of stress on body weight and pellet production between days 8–10 do not simply mirror the effects of stress on days 1–3. Furthermore, in a separate group of rats, we tested whether the effects of the final two days of restraint were dependent upon previous stress history by comparing the effects of a two day stress protocol with the interrupted protocol. Two days of restraint alone did not lead to significant effects on body weight (Fig. 3A,B; two-way repeated measures ANOVA, Treatment pattern x Day, no significant main effect of Treatment pattern p = 0.92, F(1,49) = 0.010, control n = 40, 2 day stress n = 11; no significant difference of body weight on day 10, two-tailed t-test p = 0.89, t = 0.15, df = 48). In addition, two days of restraint alone did not lead to a significant effect on adrenal gland weight normalized to body weight (Fig. 3C; two-tailed t-test p = 0.87, t = 0.17, df = 16), or raw adrenal gland weight (control 39.8±3.1 g, n = 9, stress 40.1±2.8 g, n = 9; two-tailed t-test p = 0.94, t = 0.07, df = 16). Therefore, the effects of the interrupted pattern of restraint depend upon the stress history, and do not simply mirror effects of several days of stress.

### Can differences be due to number of stress exposures?

**Figure 4:**
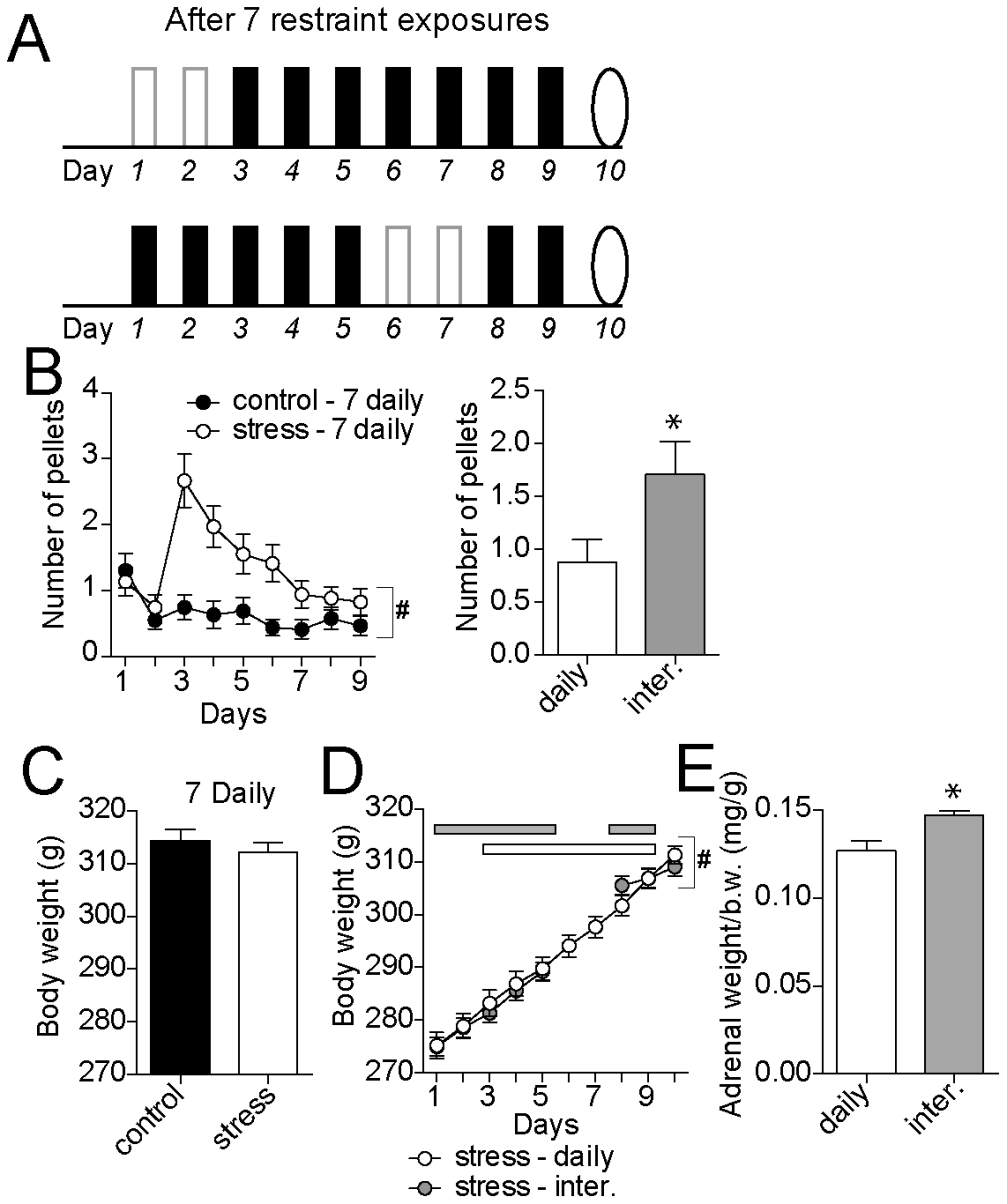
Interrupted stress has greater impact even when number of stress exposures is constant. A) It is possible that the diminished habituation after the interruption is due to a smaller total number of stress exposures. To test this, the interrupted stress pattern with 7 exposures (bottom) was compared to a daily stress pattern with 7 exposures (top). Solid bars represent stress exposure, open bars represent no stress, and oval represents the test day. B) There was significantly greater number of pellets over the course of 7 daily restraint episodes compared to their control (left) and a greater number of pellets one the final day of restraint in the 7 day interrupted pattern compared to the 7 daily pattern (right). C) The body weight at the end of the course of 7 daily pattern was not significantly different between the control andd stress group. D) The body weight of the 7 daily stress group was not significantly different than the interrupted stress group after the final episode. However, there was a significant interaction between body weight and treatment over the course of stress exposures, consistent with a difference in the pattern of weight gain. E) Adrenal gland weight (normalized to body weight) was significantly greater in the interrupted stress group compared to the equi-numbered daily restraint group. * indicates p<0.05 in post hoc comparisons after Bonferroni corrections (B, left; E). # indicates significant Pattern x Day interaction in a two-way ANOVA.

The interrupted restraint protocol leads to a more robust response during the final restraint compared to the daily restraint protocol (e.g. Fig. 1), consistent with less net habituation. Perhaps this diminished habituation is due to a fewer number of stress exposures (9 days in the daily group versus 7 days in the interrupted group). To compare interrupted and daily restraint protocols that utilize the same number of exposures to restraint, but still retain the same age across groups, two days of handling followed by 7 daily repeated stress exposures (Fig. 4A) were compared to the interrupted pattern (as above, Fig. 4A). There was marked habituation to repeated restraint over the 7 consecutive days of daily stress, measured as the number of pellets during restraint (Fig. 4B; 7 day control n = 35, 7 day stress n = 36, two-way repeated measures ANOVA, Treatment x Day, significant main effect of day p<0.0001, F(8,560) = 6.4; significant main effect of treatment p<0.0001, F(1,70) = 18.5, significant interaction p<0.0001, F(8,560) = 4.9). In addition, the total number of fecal pellets on the 7^th^ restraint episode was significantly less in the 7 day daily restraint compared to the interrupted restraint group (Fig. 4B, two-tailed unpaired t-test, p = 0.031, t = 2.23, df = 61). In the 7 day daily pattern, there was no significant main effect of treatment on body weight compared to controls (two-way repeated measures ANOVA, Treatment x Day, no significant main effect of Treatment, p = 0.39, F(1,82) = 0.75), but there was a significant interaction between day and body weight (Treatment x Day interaction, p<0.0001, F(9,738) = 5.07), indicative of divergence in the time course of weight gain, similar to the 9 day stress pattern. There was no significant difference in body weight between 7 day daily control and stress groups measured one day after the final restraint (Fig. 4C; 7 day control = 314.5±1.8, n = 40, 7 day stress = 313.2±1.4, n = 44; two-tailed unpaired t-test, p = 0.25, t = 1.18, df = 82). The 7 daily repeated restraint sessions significantly increased raw adrenal weight (7 day control = 39.3±0.6 g, n = 15, 7 day stress = 41.9±0.8, n = 18; two-way ANOVA, Treatment pattern x stress, main effect of stress p<0.0001, F(1,136) = 16.2; main effect of treatment pattern p = 0.001, F(1,144) = 11.4; significant interaction p = 0.04, F(1,136) = 4.1, 7 day control and stress significantly different, p<0.05 in post-hoc comparisons with Bonferroni corrections) and normalized adrenal weight (two-way ANOVA, Treatment pattern x stress, main effect of stress p = 0.0012, F(1,136) = 11.0; main effect of treatment pattern p = 0.0006, F(1,136) = 12.3; significant interaction p = 0.05, F(1,136) = 3.9, 7 day control and stress significantly different, p<0.05 in post-hoc comparisons with Bonferroni corrections). However, the adrenal weight of the interrupted restraint group was significantly greater than the 7 daily restraint group (Fig. 4E; p<0.05 in post-hoc comparisons with Bonferroni corrections after two-way ANOVA above). The significant differences in pellet production and adrenal gland weight between the 7 daily and the 7 interrupted stress patterns demonstrate that differences between the interrupted restraint protocol compared to the 9 day daily protocol is not due to a smaller total number of restraint sessions.

### Habituation of corticosterone response to restraint

**Figure 5:**
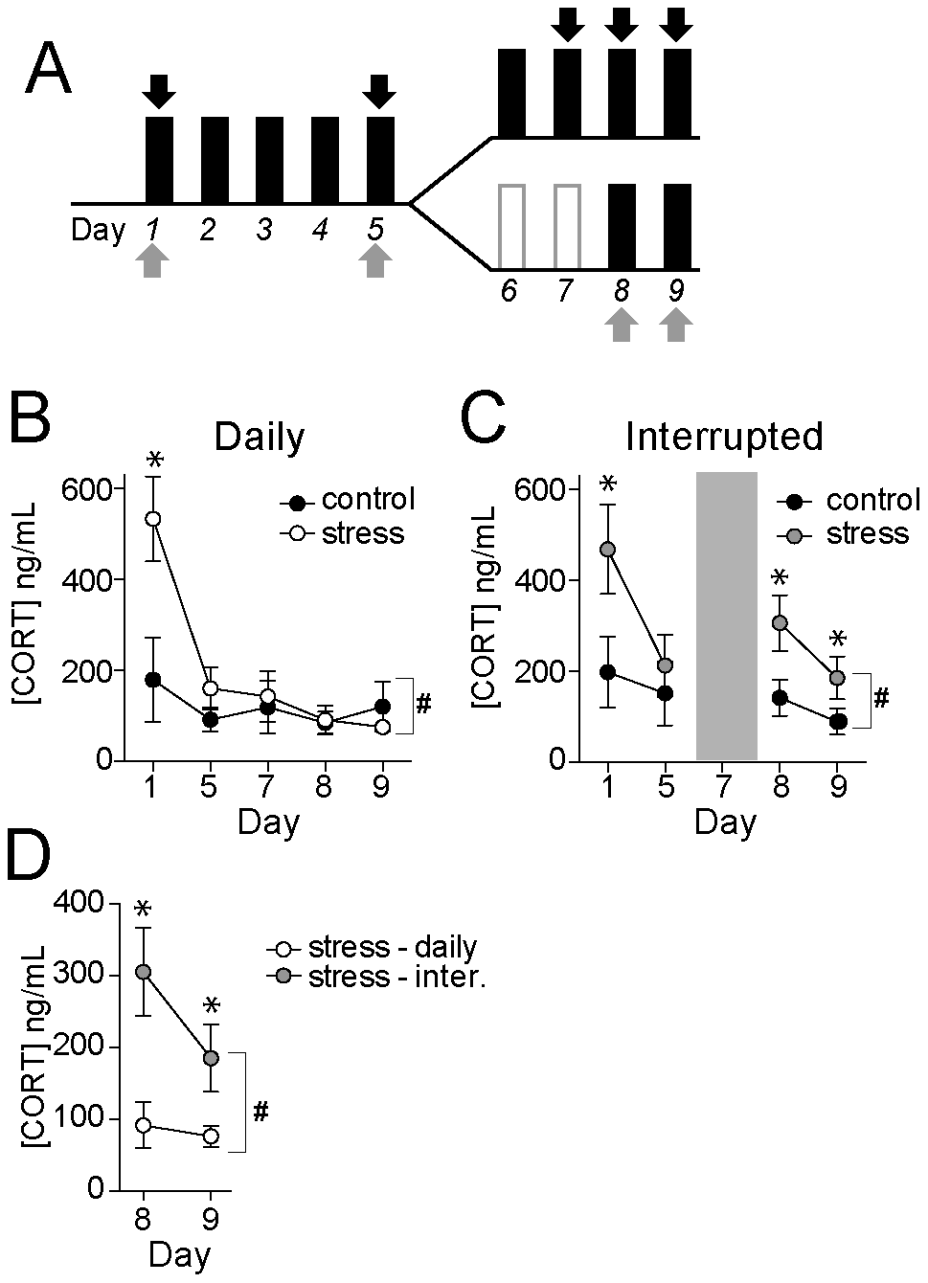
Interrupted stress leads to greater corticosterone response to restraint stress. A) Corticosterone levels were sampled on days 1, 5, 7, 8 and 9 in the daily restraint groups, and days 1, 5, 8 and 9 in the interrupted restraint groups. B) Corticosterone levels displayed habituation to control levels by the 5^th^ day of restraint in the daily restraint group. C) Corticosterone levels displayed habituation to control levels by the 5^th^ day of restraint, but displayed recovery from habituation after the interruption in the interrupted restraint group. D) When directly compared, restraint elicited greater circulating corticosterone after interruption in the interrupted restraint group compared to the daily restraint group. * indicates p<0.05 in post hoc tests after significance in a two-way repeated measures ANOVA. # indicates significant Pattern x Day interaction in a two-way ANOVA.

To measure habituation of the stress hormone response over repeated restraint, plasma levels of CORT were measured (Fig. 5A). CORT was measured from samples collected at the end of restraint or control handling sessions. Restraint stress caused a significant acute elevation of plasma CORT compared to control handling (two-way repeated measures ANOVA, Stress x Day, significant main effect of Stress p = 0.035, F(1,22) = 5.03, control n = 12, stress n = 12). However, in the daily restraint group this effect of stress was significant on the first restraint, but habituated to near control levels when measured on the 5^th^ day (Fig. 5B; no significant difference between control and daily stress on day 5, post hoc comparisons with Bonferroni corrections), and CORT remained similar to control handling when measured again on days 7–9. This is in contrast with rats that experienced interrupted restraint. There was a significant effect of stress compared to control handling on days 8–9 (i.e. post-interruption, Fig. 5C; two-way repeated measures ANOVA, Stress x Day, significant main effect of Stress p = 0.0054, F(1,40) = 8.68, control n = 6, stress n = 6; post hoc t-tests with Bonferroni corrections, p<0.05 for significance on Days 1, 8, 9). Furthermore, interrupted stress had a greater effect than daily stress on the CORT response (Fig. 5D; p<0.05 after break (days 8 and 9), post-hoc comparison with Bonferroni correction), indicative of the significant impact of an interruption in repeated stress.

### Anxiety-like behavior in the elevated plus maze

**Figure 6:**
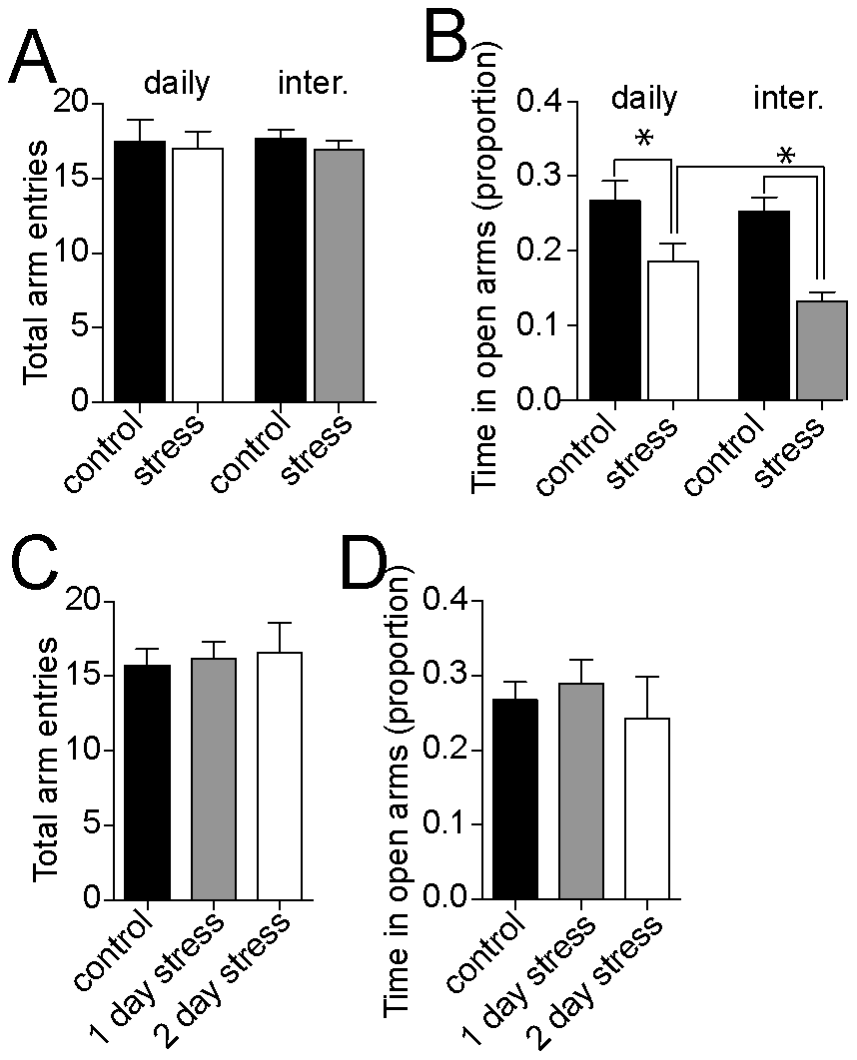
Interrupted restraint has greater impact on anxiety-like behavior. Exploration in the elevated plus maze (EPM) was measured. A) There was no significant difference in total arm entries between the 9 day daily and interrupted restraint groups, and their controls. B) Daily and interrupted restraint both led to decreased amount of time spent in the open arms. The effect of interrupted restraint stress on time in the open arms was significantly greater than the effects of daily restraint stress. C) There was no significant effect of one or two day stress exposure on total arm entries compared to controls. D) There was no significant effect of one or two day stress exposure on time in open arms compared to controls. * indicates p<0.05 in post hoc tests after two-way ANOVA.

Increased anxiety-like behavior in the EPM is a commonly observed effect of repeated stress in male rats. To test whether interrupted stress leads to greater functional impact that parallels the effects on physiological measures, behavior in the EPM was measured. There was a significant effect of stress on proportion of time in the open arms in daily (9 day) and interrupted stress groups compared to their controls (Fig. 6B; two-way ANOVA, Stress x Pattern, significant main effect of Stress p = 0.0015, F(1,281) = 10.2, significant interaction p = 0.04, F(1,281) = 4.3, control continuous n = 54, stress continuous n = 56, control interrupted n = 88, stress interrupted n = 93). Consistent with greater effects of interrupted restraint, the interrupted restraint group displayed significantly less exploration in the open arms of the EPM compared to the daily restraint group (p<0.05, post-hoc comparison with Bonferroni corrections). This indicates that interrupted stress exerts greater behavioral impact on anxiety than daily stress. There was no significant difference in total activity, quantified as total number of arm entries (Fig. 6A; two-way ANOVA, no significant main effect of Stress p = 0.42, F(1,281) = 0.64, no significant main effect of Pattern p = 0.93, F(1,281) = 0.008). In addition, there was no significant effect of single or two day stress patterns on exploration in the open arms of the EPM (Fig. 6D; time in open arms; two-way ANOVA, no significant main effect of Stress p = 0.96, F(1,105) = 0.02, no significant main effect of Pattern p = 0.51, F(1,105) = 0.44), or total arm entries (6C; two-way ANOVA, no significant main effect of Stress, p = 0.8, F(1,105) = 0.08, no significant main effect of Pattern p = 0.8, F(1,105) = 0.07).

## Discussion

The response to repeated stressors over time is dependent upon an interplay between intensity of the stressor, pattern of stress presentation and habituation to the stress. In addition, the acute response to homotypic stress is strongly attenuated with repeated exposures (i.e. habituation), while the long-lasting outcomes of the effects of stress are enhanced with repeated exposures. It has been predicted that increased homotypic habituation to acute stress predicts greater cumulative effects on longer lasting outcomes. This study tested whether the stress exposure pattern exerted parallel modification of homotypic habituation and longer lasting outcomes. The acute response to repeated stressors was quantified by struggling behavior and fecal pellet production during restraint, and CORT release in response to the stressor. The net outcomes of repeated stress exposure were measured by adrenal gland weight, body weight and anxiety-like behavior in the EPM. This study found that an interrupted pattern of repeated stress can lead to a greater effect on all of the longer lasting outcomes, despite diminished effects on habituation compared to daily stress. This was observed even if the interrupted pattern included fewer total number of stress exposures. This provides direct evidence that the pattern of exposure significantly contributes to the effects of stress, and that the link between stress habituation and outcome does not follow the predictions described above.

Similar to previous studies, repeated exposure to a relatively mild restraint stress leads to significant habituation of the acute response to that stressor [10], [15]–[19]. This was manifested as reduction in the number of pellets produced during stress, normal weight gain after an initial drop upon several days of stress, and reduced CORT response to the stress. This habituation emerged by the fifth consecutive day of repeated stress. Habituation of the stress response is expected to display a degree of spontaneous recovery after breaks [36]. This study found that a two day interruption appropriately placed in the repeated stress pattern can stall habituation and even lead to a renewal of the response to acute stress, consistent with reversal of habituation. This led to pattern-dependent differences in the habituation of the acute response to a stressor and differences in the net effects of stress on adrenal and body weight and behavior; habituation was greatest in the daily exposure pattern while the net effects on adrenal weight, body weight and anxiety behavior were greatest in the interrupted exposure pattern. Typically, habituation is more complete if a greater number of stressors are presented, and in parallel, a greater number of stressors leads to larger impact on anxiety behavior, weight and endocrine function. However, the current data indicate that this parallel may predict the response to daily, but not interrupted restraint stress.

There are several possible explanations for the greater response to restraint during the final session of an interrupted exposure compared to daily exposure. It could be due to decreased habituation as a result from a smaller number of stress exposures in the interrupted stress group. However, when the number of daily exposures is reduced to equal the interrupted exposures, there is still a greater response in the interrupted group. Further consistent with less importance of the absolute number of stress exposures, or their duration, once habituation has been achieved, 5 days of 3 hour restraint resulted in a similar degree of habituation of the CORT response as 10 days of 0.5 hour restraint [54]. A different alternative is that stressors delivered over a shorter period of time lead to greater habituation [31], [32]. However, that would not explain the increased responsiveness to restraint after interruption. The data are more consistent with a recovery from habituation during the interruption. Previous studies demonstrate that recovery from a repeated stressor does not occur after just one day [36], might not even be complete after weeks [46], [55], [56], and can be modified by stress pattern [13], [14]. We found a degree of recovery from habituation after two days of interruption, though it was not full recovery. In addition, the effects of repeated stress after the interruption do not fully recapitulate the first exposures to restraint, consistent with incomplete habituation or rapid reassertion of habituation after interruption. A different contributing factor to the reduced habituation could be the unpredictability of the stress [57]–[60]. The interruption could make the stress presentation less predictable.

Measurement of fecal boli is not a precise measure of the aversiveness of a stressor. However, in combination with the other measures (struggling and CORT), it provides an additional complementary measure. In addition, the levels of CORT measured here are somewhat higher than some previously published control values. This probably reflects the impact of our control handling procedure (placing the rat into a transport cage and bringing it into a procedure room), and that CORT sampling was not performed immediately after the lights were turned on in the animal housing room, when CORT levels are close to their lowest basal levels.

It is generally found that a greater number of stress exposures will lead to greater habituation of the response to that stressor, and a greater number of stress exposures will lead to greater behavioral outcomes. However, the relationship between habituation to repeated stress and the behavioral outcome of repeated exposure to stress is not clear. In the current study, recovery from habituation in the interrupted exposure pattern was associated with a greater impact on anxiety behavior than more extensive habituation in the daily exposure pattern. The most straightforward interpretation of these data is that the final acute response to a repeated stressor is not directly predictive of the ultimate outcome on anxiety behavior. In addition, it is usually assumed that a short break from uncontrollable stress can mitigate the cumulative effects of stress. Our results may indicate that this pattern of uncontrollable stress exposure may produce greater changes in adrenal gland weight and anxiety. Furthermore, while many humans are exposed to various forms of stress, not everyone develops the harmful manifestations of stress. The greater impact of the interrupted stress pattern may contribute to some of the variability in the emergence of post-traumatic stress disorder, anxiety and depression in humans exposed to stress.
